# Buffalo Academy of Medicine

**Published:** 1903-10

**Authors:** E. A. Bowerman


### SOCIETY PROCEEDINGS.

### Buffalo Academy of Medicine

Reported by E. A. BOWERMAN, M. D., Secretary.

*Special Meeting, Tuesday, September 8, 1903.*

The meeting was called to order at 8.50 p. m. by the president, Dr. Joseph W. Grosvenor, who announced that this was a special meeting of the Academy, called to consider a proposed ordinance to lessen accidents from 4th of July explosions and from bonfires.

As General William S. Bull, Superintendent of Police, and Edward Murphy, Assistant Chief of the Fire Department, were present by invitation, on motion the privileges of the Academy were extended to them.

Dr. Lucien Howe presented a proposed city ordinance intended to regulate the sale of fireworks and explosives and their use in the city streets, confining them to the parks, and providing penalties for violations.

This was discussed by Dr. Howe, Supt. Bull, Assistant Chief Murphy, Drs. W. D. Greene, Park, Blaauw, H. R. Hopkins, Wyckoff and Van Peyma.

Dr. Howe spoke of the number of accidents resulting each year in Buffalo from fireworks and called attention to other cities having ordinances restricting their use, naming Boston, Providence, New York, Philadelphia, Cleveland, Saint Paul and Louisville.

Supt. Bull condemned the toy pistol, toy cannon, giant firecracker and giant torpedo. He spoke of the difficulty of enforcing such an ordinance and hoped that any ordinance presented to the Common Council would be one capable of practical enforcement. He assured the Academy of the support of the Police Department in its efforts to obtain the enactment and enforcement of such a measure.

Assistant Chief Murphy spoke of the number of fires and amount of damage caused annually by fireworks. He said that a meeting of the fire commissioners, held this afternoon, commended the proposed action of the Academy and promised their coöperation.

Dr. Roswell Park spoke of the prevalence of tetanus during July and the high mortality resulting from it, the disease in nearly all cases resulting from infection of wounds received by explosives. He also spoke of the great annoyance to and damage done the sick from the noise on the 4th of July.

Dr. H. R. Hopkins called attention to the necessity of procuring the coöperation of other influential bodies, such as the Chamber of Commerce, in urging the passage of such a measure.

It was moved and seconded that the Academy of Medicine heartily approve the enactment of an ordinance restricting the sale and use of fireworks in Buffalo and that a committee of five be appointed to coöperate with other bodies in urging its enactment. Carried.

The President appointed Drs. Lucien Howe, Walter D. Greene, Roswell Park, Eugene A. Smith and Peter W. Van Peyma.

On motion a vote of thanks was tendered Supt. Bull and Assistant Chief Murphy for their presence and promised support. Adjourned at 9.50 p. m.

Attendance, 30.

